# Editorial: Axonal growth in normal and pathological conditions

**DOI:** 10.3389/fnmol.2022.1107999

**Published:** 2022-12-13

**Authors:** Takao Omura

**Affiliations:** Department of Orthopaedic Surgery, Hamamatsu University School of Medicine, Hamamatsu, Japan

**Keywords:** axonal regeneration, cAMP, lecticans, let-7-7a-5p, PLPPRs, Ctcf, Yy1

A central problem in clinical neuroscience is that the regenerative capacity of the injured adult mammalian central nervous system (CNS) is extremely limited, which usually leads to permanent neurological deficits. The reason there is no regeneration in the CNS after the injury is because the injury does not increase the intrinsic growth capacity of the injured neurons, their growth state remains unchanged or even suppressed and they continue to respond to inhibitory cues in the CNS (Fournier et al., 2001; Filbin, 2003; Fitch and Silver, 2008). Patients with severe CNS injuries such as complete spinal cord injuries (SCI) can suffer permanent deficits despite the surgical treatment, as such procedures are mainly limited to decompression and stabilization of the site of injury, but not the rewiring of the network. For the axons to regrow and restore any function, the CNS neurons requires reactivating injured neurons' intrinsic growth state and enable growth in an inhibitory environment.

Adult neurons in the peripheral nervous system (PNS) can be switched or transformed, however, into an actively growing state. For dorsal root ganglion (DRG) sensory neurons, preconditioning the sensory neurons by applying peripheral nerve injury both increases axonal growth and partially overcomes myelin inhibition, acquiring the novel capacity to regenerate in the CNS (Neumann and Woolf, 1999). The initiation of regeneration requires the neuronal cell body to alter its macromolecular synthesis patterns in response to axonal injury, including up-regulation of cytoskeletal proteins, cell adhesion and axon guidance molecules, trophic factors, and their receptors; collectively constituting an expression of a complex set of what has been termed regeneration-associated genes (RAGs). Among the pathways, manipulating cytoplasmic cAMP signaling has been proven to efficiently promote injury repair and functional recovery (Neumann et al., 2002). Zhou et al., summarize that elevated neuronal cAMP levels not only enhance intrinsic axonal growth during the development process but also promote neural survival and regeneration after injury. Interestingly, cAMP has also been shown to regulate the function of glial cells and interact in complex ways with other signals generated by SCI, including macrophage/microglia polarization, astrocyte activation, and oligodendrocyte differentiation. In the mammalian CNS, the reported downstream effectors of cAMP include protein kinase A (PKA), exchange protein directly activated by cAMP (Epac), and cyclic nucleotide-gated channel. Epac is expressed as two isoforms, Epac1 and Epac2, which exhibit a different expression pattern in vertebrates. Epac1 expressed in early neurons contributes to primary axon development, and Epac2 expressed in mature neurons modulates the dendrite stability and outgrowth. cAMP is also responsible for axonal guidance as low cAMP concentrations in the growth cone result in repulsion in the presence of guidance cues such as MAG and Netrin-1, but high concentrations result in attraction. Given the multi roles of cAMP in the CNS, cAMP is a potential candidate for CNS regeneration impairments.

Mesenchymal stem cells (MSCs) and extracellular vesicles (EVs) are promising treatments for SCI. In the review by Wang et al., they report the role of micro-RNA let-7-7a-5p derived from MSCs. EVs from bone marrow-derived MSCs promoted the differentiation of neural stem cells (NSCs) into the neurons and outgrowth of neurites that are extending into astrocytic scars in SCI rats. Both BMSC-extracellular vesicles and let-7a-5p promoted the differentiation of neural stem cells into neurons and the inhibition of let-7a-5p attenuate the BMSC extracellular vesicle-induced effects through activation of TGF-beta/Smad signaling pathway.

Neuronal plasma membrane proteins orchestrate neuronal differentiation, growth and plasticity in the developing and adult nervous system and the review by Fuchs et al. present how plasma membrane proteins phospholipid phosphatase-related proteins (PLPPRs) functions in filopodia formation, axon guidance and branching during nervous system development and regeneration, as well as in the control of dendritic spine number and excitability. PLPPRs govern neurite and/or branch formation in a PI(3,4,5)P3-dependent manner and axon guidance is regulated though interaction with TRIM9 and TRIM67, which are E3 ubiquitin ligases that localize to filopodia tips and function in axon projection. Although the interaction of PLPPR with LPA signaling is yet to be elucidated, PLPPR remains an intriguing target in CNS regeneration.

Among Nogo, MAG, and oMGP, chondroitin sulfate proteoglycans (CSPGs) are believed to be the major inhibitory cues in the CNS. Interestingly, review by Zhang et al., shows that lampreys can regenerate their axons after SCI increase CSPGs expression. Lecticans are the major component of CSPG family and four types of lamprey lecticans (A, B, C, and D) were found in glia and neurons. Of these lecticans, upregulation of lectican D was particularly strong and persistent, at least to 10 weeks post-SCI, when many axons are already regenerating into the scar, suggesting that post-SCI, some CSPGs, particularly lectican D, might actually promote axon growth or guidance and this may change the view for overcoming inhibitory environment.

Despite the regenerative capability of PNS neurons, this capacity is still not enough to restore meaningful function after peripheral nerve injury, depending on the site, magnitude, and the type of injury. In order to achieve meaningful function after peripheral nerve injury, the regenerating axons must reach their target muscle before the distal Schwann cells and the muscles degenerate. There are three strategies to promote PNS regeneration. (1) Acceleration of neuronal intrinsic growth, (2) prevention of Schwann cell degeneration apoptosis, and (3) prevention of muscle irreversible degeneration. Using RNA sequencing at different time points in cultured dorsal root ganglia neurons. Avraham et al. discovered that combination of overexpressed transcription factors Ctcf and Yy1 significantly enhances axonal growth by over 50% but interestingly, does not show significant growth when overexpressed independently. For traumatic peripheral nerve injuries, the tools we have for nerve repair is limited to direct nerve suture, autologous or artificial nerve graft for nerve defects and nerve transfers. However, the procedures are limited by the donor nerves, so as a surgeon, we truly hope for this advancement made in basic science to be transitioned to clinical practice. For patients with limited function, small improvement can bring huge changes.

## Author contributions

The author confirms being the sole contributor of this work and has approved it for publication.
